# HybridMine: A Pipeline for Allele Inheritance and Gene Copy Number Prediction in Hybrid Genomes and Its Application to Industrial Yeasts

**DOI:** 10.3390/microorganisms8101554

**Published:** 2020-10-09

**Authors:** Soukaina Timouma, Jean-Marc Schwartz, Daniela Delneri

**Affiliations:** 1 Manchester Institute of Biotechnology, Faculty of Biology Medicine and Health, University of Manchester, M1 7DN Manchester, UK; 2 Division of Evolution and Genomic Sciences, School of Biological Sciences, Faculty of Biology Medicine and Health, University of Manchester, M13 9PT Manchester, UK

**Keywords:** hybrids, prediction, parental alleles, orthologs, yeast

## Abstract

Genome-scale computational approaches are opening opportunities to model and predict favorable combination of traits for strain development. However, mining the genome of complex hybrids is not currently an easy task, due to the high level of redundancy and presence of homologous. For example, *Saccharomyces pastorianus* is an allopolyploid sterile yeast hybrid used in brewing to produce lager-style beers. The development of new yeast strains with valuable industrial traits such as improved maltose utilization or balanced flavor profiles are now a major ambition and challenge in craft brewing and distilling industries. Moreover, no genome annotation for most of these industrial strains have been published. Here, we developed HybridMine, a new user-friendly, open-source tool for functional annotation of hybrid aneuploid genomes of any species by predicting parental alleles including paralogs. Our benchmark studies showed that HybridMine produced biologically accurate results for hybrid genomes compared to other well-established software. As proof of principle, we carried out a comprehensive structural and functional annotation of complex yeast hybrids to enable system biology prediction studies. HybridMine is developed in Python, Perl, and Bash programming languages and is available in GitHub.

## 1. Introduction

Natural or artificial hybridization between strains or species is a common phenomenon that occurs in almost all sexually reproducing group of organisms, including bacteria, yeast, plants, and animals [1]. It has been established that there is at least 25% of plant species and 10% of animal species involved in hybridization with other species [2].

**Figure 1:**
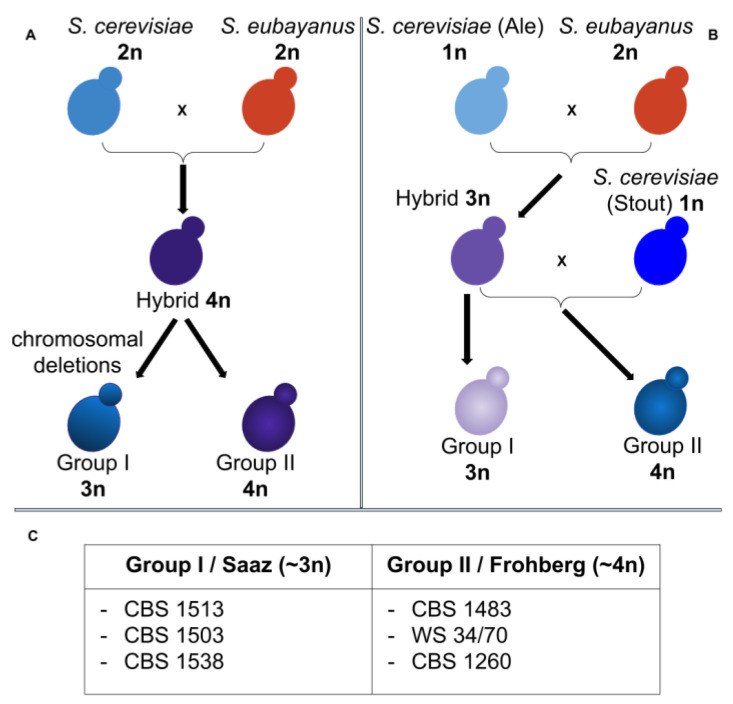
Proposed origins of Group I and II *Saccharomyces pastorianus*. (**A**): A hybridization event occurred between a diploid *S. cerevisiae* and a diploid *S. eubayanus*, followed by a different amount of genome reduction. (**B**): A hybridization event between a haploid *S. cerevisiae* and a diploid *S. eubayanus* lead to a triploid progenitor. Subsequently, a second hybridization event occurred between the triploid progenitor and a haploid *S. cerevisiae*. (**C**): Strains belonging to the Group I and II (adapted from Alsammar et al., 2020).

Hybridization between yeast species is an attractive example of how a specific combination of traits from parental species can favor adaptation to harsh fermentative conditions. *S. pastorianus* is an allopolyploid sterile hybrid of the mesophilic *Saccharomyces cerevisiae* and the cold tolerant *Saccharomyces eubayanus*. These aneuploid hybrid strains have the beneficial properties of both parents, such as a strong ability to ferment at low temperature (bottom-fermenting lager yeast) and under stressful conditions, such as anaerobiosis, high hydrostatic pressure and high gravity sugar solutions [3]. *S. pastorianus* strains carry multiple copies of *S. cerevisiae*-like, *S. eubayanus*-like and hybrid-gene alleles, which encode for different protein isoforms. This may lead to agonistic or antagonistic competition for substrates and varying biochemical activities resulting in novel phenotypes and unique cellular metabolism. Moreover, different chimeric protein complexes can be established in the hybrids producing a plethora of phenotypes [4]. In these environmental conditions, the fermenting process produces complex metabolites that lead to unique flavors and aromas, appreciated in beer beverage. *S. pastorianus*, originally named *Saccharomyces carlsbergensis*, has been isolated from lager fermentation environments [5]. The analysis of transposon sequence distribution in the genome of different *S. pastorianus* strains suggested the presence of two genomically distinct groups [6], which may have arisen from different hybridization events (Figure 1) [7]. One theory supports the hypothesis that an initial hybridization event occurred between a diploid *S. cerevisiae* and a diploid *S. eubayanus* (Figure 1A), leading to a tetraploid hybrid progenitor, which evolved to give the Group II strains. This progenitor in parallel underwent chromosomal deletions of the *S. cerevisiae* sub-genome, leading to the Group I strains [8]. Another hypothesis states that an initial spontaneous hybridization event occurred between a haploid *S. cerevisiae* strain and a diploid *S. eubayanus* strain during the Middle Ages (Figure 1B) [9]. This event led to a progenitor of the Group I strain, which evolved through further reduction of the *S. cerevisiae* genome content, to produce the extant Group I strains, which are approximately triploid in nature. The *S. cerevisiae* parent of the Group I yeasts is related to yeast strains used for Ale beer production in Europe. In parallel, the Group I progenitor strain underwent a second hybridization event with a *S. cerevisiae* strain related to Stout fermentation and evolved to give Group II strains with an approximate tetraploid genome. It appears that the Group II strains have 2–3 times more *S. cerevisiae* genomic content than the Group I strains. Therefore, Group II yeasts have a rather complex genome containing Ale-like (*S. cerevisiae*), Stout-like (*S. cerevisiae*, British Isles) and lager-like (*S. eubayanus*) gene content. The geographical and brewery location are linked to the grouping of *S. pastorianus* strains. The Group I encompasses both Saaz-type strains from Czech Republic breweries and Carlsberg type strains from Denmark breweries (Figure 1C). The Group II, referred as Frohberg-type, includes strains found in two Canadian breweries, in Heineken and Oranjeboom breweries in the Netherlands, and in non-Carlsberg breweries in Denmark (Figure 1C) [10].

Several lineages of *S. eubayanus* have been isolated from Nothofagus trees in Patagonia, and more recently in East Asia (Tibet) [11], while *S. cerevisiae* was isolated in Europe. The silk road, which connected Asia to Europe for trading purposes, can explain how the hybridization occurred between those two species. Before the discovery of *S. eubayanus*, the non-*S. cerevisiae* portion of the genome of *S. pastorianus* was considered as being *Saccharomyces uvarum* and/or *Saccharomyces bayanus* genome, which are closely related to *S. eubayanus* [12]. Moreover, *Saccharomyces* group yeast went through a whole genome duplication event (WGD), about 100 million years ago [13]. The WGD has important consequences as the organism doubles its genetic content leaving one copy of each gene free from constraints and able to evolve. Although the majority of paralogous will simply accumulate mutations and become pseudogenes (non-functionalization), some can acquire new functions (neo-functionalization) or can share the original function between them (sub-functionalization). Therefore, orthology relationships resulting from the WGD events are complicated as it leads to a 2:1 synteny relationship between genomic regions in post-WGD and non-WGD species [14]. Orthologs are homologs that arose from a specific genetic locus in the last common ancestor and evolved in the descendants after speciation events. Yeast paralogs created via WGD are called ohnologs, while the one derived small-scale duplication events are more generally referred as paralogs [15]. We know now that almost all eukaryotic sequences show signs of ancient duplications, either WGDs or segmental duplications [14].

Mining the complex genome of these hybrids is therefore difficult. *Saccharomyces pastorianus* popularity is growing as one of the world’s most important industrial organism [16] and several R&D departments of the brewery industries worldwide are now focusing on strain improvement [17]. The strains *S. pastorianus* CBS 1503 (known as *Saccharomyces monacensis*), CBS 1513 (known as *S. carlsbergensis*), CBS 1538 and WS 34/70 (known as *weihenstephan* strain) [18,19,20] used in the beer market, have been recently sequenced and assembled, but no functional annotation has been published, hampering biotechnological processes. It is in fact becoming essential to develop analytical and predictive tools to allow tailor-made improvements of specific yeast traits such as ethanol tolerance, maltose utilization, and flavor profile. Established functional annotation tools such as Blast2GO [21] are computationally intensive and come with a costly license. eggNOG-mapper [22] is not ideal for hybrid genomes as it transfers annotations by searching orthologs in a wide taxa group, hampering the discrimination of parental alleles. Finally, both Blast2GO and eggNOG-mapper are not designed to take into account aneuploidy and paralogous genes are discarded. Here, we developed HybridMine, a specific open-source computational tool for annotating hybrid genomes by predicting parental alleles including paralogs. HybridMine is user-friendly, extremely fast, and reliable to predict orthology and gene families. As proof of principle, the genome of four *S. pastorianus* hybrid strains have been functionally annotated with our tool. A significant correlation between predictive and expected parental allele content has been observed, confirming the reliability of HybridMine.

## 2. Materials and Methods

### 2.1. HybridMine Pipeline Architecture

**Figure 2:**
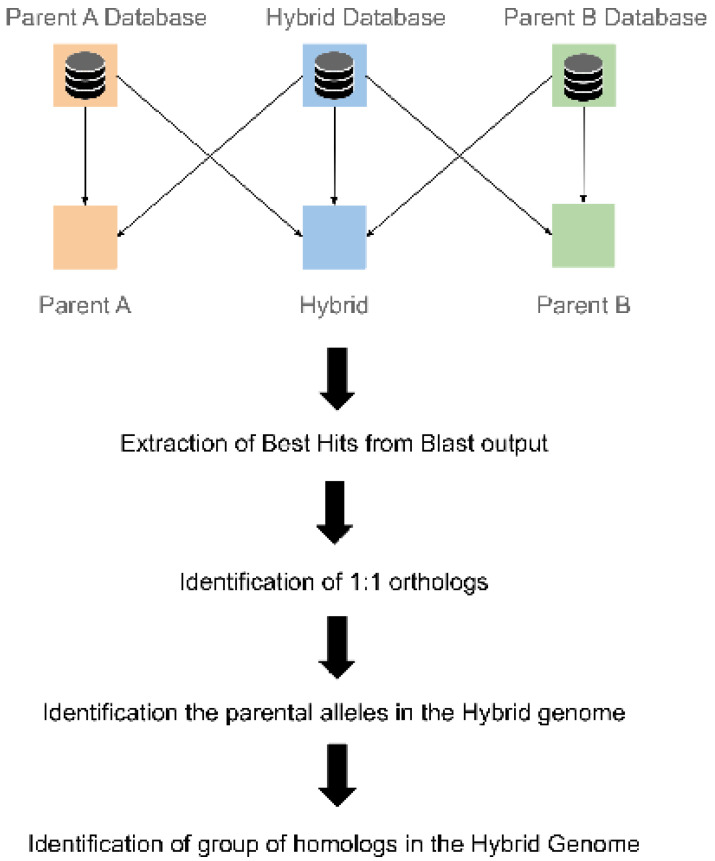
HybridMine pipeline. The execution steps are indicated by the bold arrows. Firstly, three Blast databases are created for the hybrid and the parental species. Genes of the hybrid are blasted against all the databases and the parents are also blasted against themselves. Secondly, best hits are extracted from each Blast output and are used in the next step to determine the one-to-one orthologs between the hybrid and the parents, and the homolog genes in each genome. Parental alleles in hybrid genome are then identified from the one-to-one orthologs. Finally, the homolog genes in the hybrid are annotated with the parental alleles.

The script for the HybridMine architecture has been developed in Bash language. The alignments have been done using BLAST 2.6.0+ program [23]. Information were extracted from the BLAST outputs using regular expressions in Perl language (Perl Foundation, Perl programming language available at https://www.perl.org/). To identify orthologs, the parental alleles and group of homologs in the hybrid genome, three scripts have been developed in Python 3.6 (Python Software Foundation. Python version 3.6 is available at http://www.python.org). A diagram summarizing the pipeline architecture is presented in Figure 2. HybridMine has been developed, tested, and optimized in a Linux environment (Ubuntu 18.04.4 LTS), on an Intel^®^ Core™ i9-7900X CPU @ 3.30GHz × 20 machine. HybridMine is available at https://github.com/Sookie-S/HybridMine.

### 2.2. Validation Dataset

The genome assemblies for the strains *S. pastorianus* CBS 1503, CBS 1513, CBS 1538, and WS 34/70 [20] were downloaded from National Center for Biotechnology Information (NCBI) and were annotated by HybridMine. The *S. cerevisiae* S288C genome was used as a reference to annotate the *S. cerevisiae*-like genome content in the *S. pastorianus* strains. The last released *S. cerevisiae* genome was downloaded from the Saccharomyces Genome Database (SGD). The *S. eubayanus* FM1318 strain was used as a reference to annotate the *S. eubayanus*-like genome content in *S. pastorianus*. Its genome assembly and annotation provided by the Tokyo Institute of Technology was taken from the NCBI database. Complementary information about the four *S. pastorianus strains* and the link to their repository are given in Supplementary Table S1. 

The Yeast Genome Annotation Pipeline (YGAP) [24] was used to predict the position of potential open reading frames (ORFs) and tRNAs in these strains. YGAP is a structural annotation system that uses homology and synteny information from other yeast species present in the Yeast Gene Order Browser database, based on the hypothesis that the genes intron/exon structure is conserved through evolution (two orthologous genes might have a similar intron/exon structure). The pipeline was chosen as it is suitable for species that went through the Whole-Genome Duplication event.

The Bio::Tools::GFF module in the BioPerl bundle has been used to convert YGAP output GenBank files to GFF3 files for the four *S. pastorianus* strains. A Python 3.6 script was developed to replace the fake gene IDs (generated by YGAP) by the parental gene name predicted by HybridMine.

The *S. cerevisiae*-like and *S. eubayanus*-like genes sharing the same function in the *S. pastorianus* genome were identified by searching for one-to-one orthologs between the *S. cerevisiae* S288C and *S. eubayanus* FM1318 parental strains, using Python 3.6. The Venn diagrams were generated using the Python 3.6 matplotlib_venn package.

### 2.3. Statistical Analysis

The difference between the HybridMine predicted and expected number of parental alleles obtained in four *S. pastorianus* strains was tested using the Chi square test. A *p*-value of less than 0.01 was considered statistically significant. Statistical analysis was performed using Python 3.6 packages Scipy and Stats models.

### 2.4. Homologs Validation

HybridMine homologs prediction was validated by verifying the accuracy of its prediction for *S. cerevisiae* S288C. A list of paralog genes in *S. cerevisiae* S288C genome was downloaded from the *Saccharomyces* Genome Database. This dataset was curated by removing the duplicated paralogs using Python 3.6 and enriched by adding paralog genes coming from small scale duplication identified in Katju et al., 2009 study [15]. Specific features of the HybridMine pipeline were used to predict young paralogs in *S. cerevisiae* genome. A local alignment (Blastn) was carried out on *S. cerevisiae* S288C genes against itself in order to get the best hits. Best bidirectional hits have been selected as paralogs (“*paralogs.py*” script) and grouped when they share one paralog (“*prediction.py*” script). Data mining has been performed to collect annotations on the *S. cerevisiae* S288C paralog genes identified by HybridMine. A Python 3.6 script that uses the Representational State Transfer (REST) Application Programming Interface (API) of UniProt database [25] was developed to query and access its data.

To validate HybridMine outputs of orthologs and homologs prediction, alignment and phylogenetic reconstructions were performed using the function “build” of ETE3 v3.1.1 [26] as implemented on the GenomeNet (https://www.genome.jp/tools/ete/). A distance-based tree was inferred with the BioNJ algorithm [27] using PhyML v20160115 [28] ran with model GTR and parameters: -f m–bootstrap -2–alpha e -o lr –nclasses 4–pinv e. Branch supports are the Chi2-based parametric values return by the approximate likelihood ratio test.

### 2.5. Benchmark Validation of HybridMine

We compared the results and performances of HybridMine to predict parental alleles, one-to-one paralogs, and group of homologs with two well-established annotation tools, which use BLAST algorithms, Blast2GO [21], and eggNOG-mapper [22]. We used the trial-version of Blast2GO. All the tools have been run on the same Intel^®^ Core™ i9-7900X CPU @ 3.30 GHz × 20 computer.

### 2.6. Code Availability

The computational resources described in this paper and the genome annotations are available in GitHub (https://github.com/Sookie-S/HybridMine).

### 2.7. Data Availability

HybridMine has been used to predict the parental alleles and paralogs in four S. pastorianus strains: CBS 1503 (known as S. monacensis), CBS 1513 (known as S. carlsbergensis), CBS 1538 and WS 34/70 (known as weihenstephan). The predictions are downloadable from the Supplementary Files S1–S4. The genome annotations generated in GFF3 format are available in GitHub (https://github.com/Sookie-S/HybridMine/tree/master/Annotation_files) and can also be downloaded from Dryad (https://doi.org/10.5061/dryad.3n5tb2rdf).

## 3. Results

### 3.1. Allele Inheritance Prediction Pipeline

**Figure 3:**
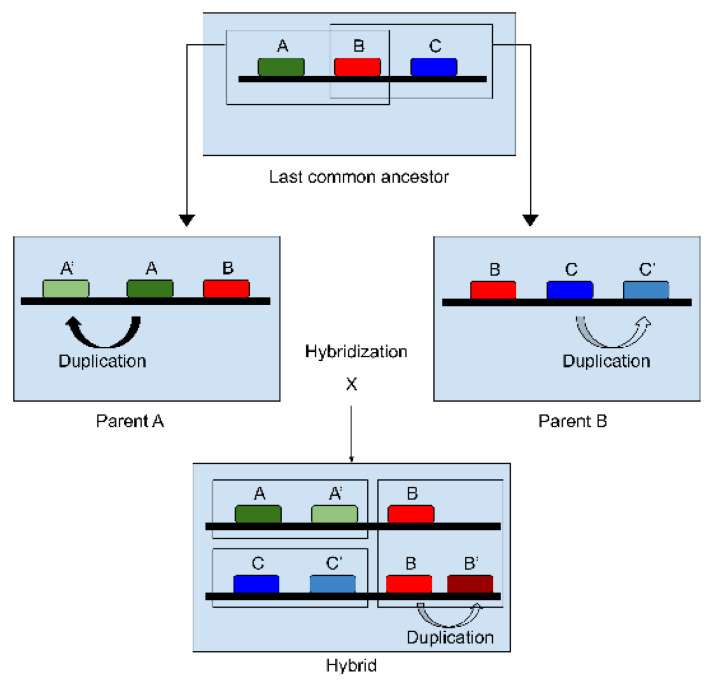
Hybrid homolog genes hypothetic origins: Parent A and parent B can share one-to-one orthologs (i.e., gene B in red box) inherited from their last common ancestor after speciation. Parent A and parent B evolved and accumulated paralogs from duplication events (i.e., genes A and A’ in parent A, and genes C and C’ in parent B). After hybridization, the hybrid genome can inherit paralogs from each parent (i.e., A and A’, C and C’), but also orthologs (i.e., gene B) that share a high percentage of homologs and same function. Duplication events occur in the hybrid genome (i.e., B and B’).

**Figure 4:**
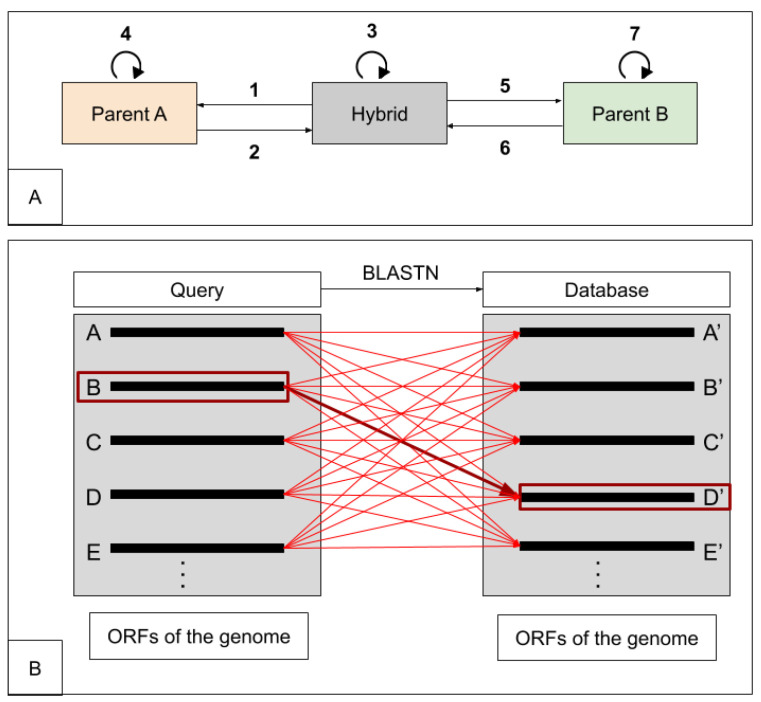
(**A**): The hybrid genome is blasted against the parental genomes (line 1 and 5) and reciprocally (line 2 and 6) to identify the orthologs. Each genome is searched against itself (line 3, 4, and 7) in order to get the best paralogs when they exist. (**B**): each ORF of the query genome is blasted against the database genome (red lines). When two ORFs are shown as reciprocal best hit (bold red arrows), the so called “best bidirectional hit” is identified. For example, ORF B (boxed in red) of the hybrid has as best hit the ORF D’ (boxed in red) of the parent A and vice versa.

We developed a pipeline based on homology search using BLAST algorithms to identify the parental alleles in hybrid organisms. Divergence in orthologous genes is only considered to be due to speciation which allows direct functional inference. In the hybrid genome, the group of homolog genes include paralogs coming from small scale duplication that occurred after the hybridization event, but also orthologs inherited from the parental genomes, and paralogs inherited from the parental genomes (Figure 3). HybridMine searches for these groups of homologs in the hybrid genome. The main execution file (*pipeline.sh*) launching the pipeline is run from a Unix terminal and requires as inputs at least three FASTA files containing all the ORFs sequences of the hybrid strain to annotate, the first parent (i.e., Parent A) and the second parent (i.e., Parent B), up to four parents. Initially, BLAST databases are built to match query genomes. To determine the best hits, nucleotide-nucleotide Blast commands are run stringently (i.e., expectation value threshold for saving hits set at 0.05, default 10). Seven different blastn commands have been run to identify best bidirectional hits and homologs (Figure 4A). For each run, the best alignments are written in a blast output file. Subsequently, a Perl script (*blast_parser.pl*) parses the output blast files and employs regular expressions in order to catch the query’s best hits in the database. The parser catches the e-value, the associated sequence identity and gap percentage for each best hit. As output, the script generates files containing the best hits for each gene in the queries. A Python script (*homologs.py*) identifies the homologs in each genome. Then a Python 3.6 script (*orthologs.py*) determines the 1:1 orthologs by finding the best bidirectional hits between the hybrid and the parents. The script transforms each ORFs of the query genome into a Python object, defined by the following attributes: ID, best hit, best hit e-value, best bidirectional hit, and best bidirectional hit e-value. A best hit is only considered if it shares more than 80% identity with the ORF of the query. A best bidirectional hit occurs when two ORFs are reciprocally found as best hits (Figure 4B). The two output files generated (containing the one-to-one orthologs between the hybrid strain and the parent A, and those between the hybrid and the parent B) are then used as input in the next step of the pipeline. The last Python script (*prediction.py*) determines which is the most likely parental allele the 1:1 ortholog evolved from. In the instance where a hybrid’s ORF has both a 1:1 ortholog in parent A and in parent B, the one that shares the highest percentage of identity is kept as parental allele. The only case in which a parental origin of an ortholog cannot be assigned is when the sequence of the orthologs in parent A and parent B are the same. For example, this can occur for tRNAs since they are extremely conserved and share 100% identity between the two parents. Once the origin of the alleles in the hybrid are identified, they are given the ID of the parental gene. This script determines the groups of homolog genes, including paralogs, ortholog parental alleles inherited, and paralog parental alleles inherited, in the hybrid genome. When two pairs of homologs share one gene in common, they are grouped in as common homologs.

### 3.2. HybridMine Usage

**Figure 5:**
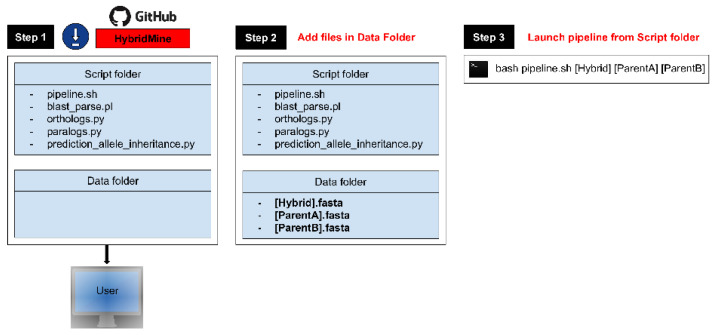
HybridMine usage. Step 1: Users to download HybridMine from its GitHub repository. Step 2: Users to add the three input fasta files required (ORFs of the hybrid organism, parent A and parent B) in the Data directory. Step 3: Users to run the main execution file “*pipeline.sh*” from the Script directory in a Unix terminal.

HybridMine package is composed by two folders, “Script” and “Data”, which needs to stay co-located in the same directory when downloaded (Figure 5, Step 1). The user places in the Data directory the 3 FASTA format files, containing the ORFs of the hybrid to annotate, the ORFs of the parent A and the ORFs of the parent B (Figure 5, Step 2). Subsequently, the user launches the main execution file (*pipeline.sh*) from the “Script” directory, specifying as input the FASTA files containing the genes sequence of the hybrid to annotate and its parental organisms (i.e., command line: bash pipeline.sh (Hybrid.fasta) (ParentA.fasta) (ParentB.fasta); see Figure 5, Step 3). HybridMine can accept up to four parental strains as input. The documentation is presented in GitHub (https://github.com/Sookie-S/HybridMine/blob/master/README.md).

### 3.3. Paralogs Validation

Since there are no hybrid genomes with both parental alleles mapped and annotated, either within yeast or within other eukaryotic organisms, we used the *S. cerevisiae* S288c strain to test our tool to predict homologs and paralogs. HybridMine predicted 257 groups of homologs in *S. cerevisiae* S88C genome. Strikingly, HybridMine has accurately grouped homolog genes belonging to a same family (see Supplementary Table S2). All the genes isoforms coding for histones, 40S and 60S ribosomal proteins have been accurately predicted as paralogs, as well as enzymes isoforms such as the serine/threonine-protein kinase *PSK1* with *PSK2*, and NADP-specific glutamate dehydrogenase 1 (NADP-GDH 1) with NADP-GDH 2, among many others. Interestingly, some groups of homologs predicted by HybridMine contain an uncharacterized gene whose family can be therefore inferred. For example, YIR043C of unknown function has been grouped with the *COS* chitosan oligosaccharide family (9 genes *COS1* to *COS9*).

We then mapped known paralogs from small scale duplications (SSD) to validate HybridMine predictions. There are 30 pairs of SSD paralogs reported in SGD. This list has been enriched with 11 supplementary SSD paralogs reported in Katju et al., 2009. HybridMine accurately predicted 39/41 of these SSD paralogs and omitted two of them. The undetected pair of paralogs, YCR094W/YNL323W and YIL029C/YPR071W have diverged too much and therefore were not detected by Blastn. 

Regarding duplicates that arose from the ancient WGD event, out of the 550 pairs of ohnologs reported by SGD to be present in the *S. cerevisiae* genome, 455 pairs have diverged significantly. The remaining 95 pairs, which display high sequence homology and therefore can be detected by Blastn, were all identified by HybridMine.

### 3.4. Benchmark Evaluation

Blast2GO is a bioinformatics platform for functional annotation and analysis of genomic datasets, available under a costly license. Blast2GO uses Blast algorithms to search for homologous genes in any Blast database, and also offers to the user to create a restrained database. eggNOG-mapper is a tool for genome-wide functional annotation through orthology assignment. It searches for one-to-one orthology, which permits a higher precision than traditional homology searches (Blast2GO), as it prevents transferring annotations from close paralogs. However, eggNOG-mapper does not restrain the search to specific parental genomes. 

Here, we compared the results and performances of HybridMine with these two well-established annotation tools which use BLAST algorithms.

We did the testing using the genome of *Saccharomyces pastorianus* WS34/76, the largest hybrid in this study (11,265 ORFs). This strain is aneuploid, having a nearly tetraploid genome, and it contains a high number of paralogs.

Blast2GO offers the option to build a specific BLAST database that can be employed to align the ORFs of the genome to be annotated. This database was built by using the parental genomes *S. cerevisiae* S288C (reference genome) and *S. eubayanus* FM1318 (reference genome). As an output, Blast2GO then provided the best homolog in the parental database for the *S. pastorianus* genes. Blast2GO predicted 5373 *S. cerevisiae*-like genes and 6148 *S. eubayanus*-like genes. We found that in this case the paralogs are always annotated with the same parental homolog as best hit, since Blast2GO does not take into account the aneuploid nature of the species to annotate. Such output is not useful to understand the evolutionary history of paralogs in hybrid species.

eggNOG-mapper only offers the option to restrain the Blast database at the taxonomic level, encompassing a wider and mostly irrelevant range of species. For benchmark purposes, we chose the *Saccharomycetaceae* family, which includes the genus *Saccharomyces*. In EggNOG-mapper we set the parameters to narrow the annotation only to one-to-one orthology. This would be the closest strategy to the one followed by HybridMine. As output, it predicted 9997 *S. cerevisiae*-like genes (98% of the hybrid genome) and inferred the remaining annotation from 23 unrelated yeast species. Strikingly, the tool was not able to predict any gene inherited from the *S. eubayanus* parent. Overall, EggNOG-mapper resulted to be very inaccurate for predictions of hybrid genome.

Performance wise, HybridMine was by far the fastest of the three tools. The execution time was 0 min 32 s for the annotation of *S. pastorianus* WS34/76 (sample size of 11,265 genes) compare to 9 min 40 s of Blast2GO, and 101 min 10 s of eggNOG-mapper. Overall, HybridMine is at least 15 times faster than available software and is currently the only free and open source tool able to predict accurately parental alleles and homologs (including paralogs), in hybrid aneuploid genomes. The features of the comparison for the three tools are reported in Table 1.

### 3.5. Application of HybridMine to Annotate S. pastorianus Hybrids and Validation of Outputs

**Figure 6:**
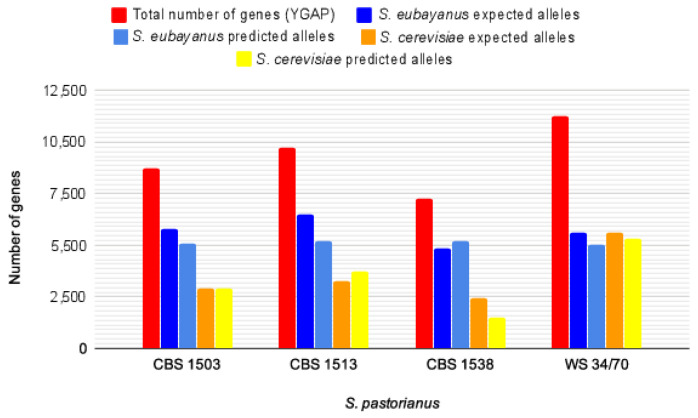
Histogram showing the amount of predicted versus expected *S. cerevisiae*-like and *S. eubayanus*-like genome content present in *S. pastorianus* strains CBS 1503, CBS 1513, CBS 1538, and WS 34/70.

As proof of principle, HybridMine was used to functionally annotate four *S. pastorianus* hybrid strains. The tool predicted approximately two-thirds *S. eubayanus*-like content and one-third *S. cerevisiae*-like content in the *S. pastorianus* hybrids CBS 1503, CBS 1513, and CBS 1538 belonging to the Group I; and approximately one-half *S. eubayanus*-like content and one-half *S. cerevisiae*-like content in the hybrid WS 34/70 that belongs to the group II (Table 2). Such predictions correlate with the expected genome content for these hybrids, approximately triploid and tetraploid for Group I and Group II strains, respectively (Figure 6). Lastly, we have generated GFF3 genome annotation files for the four *S. pastorianus* strains, which contain the parental gene IDs attributed by HybridMine.

We determined whether the difference between the observed and the expected frequencies of the *S. eubayanus*-like and *S. cerevisiae*-like alleles was statistically significant in these four *S. pastorianus* strains. Here, the null hypothesis is that there is no association between expected and observed data. We chose the value of 0.01 for the alpha risk to reject the null hypothesis when it is actually true. We obtained a chi square value of 865.44, which is superior to the critical value 18.48 of this test, and a *p*-value of 1.3 × 10^−182^, which is inferior to the risk alpha and approximal to zero. Hence, the values predicted are significantly concordant with the values expected.

**Figure 7:**
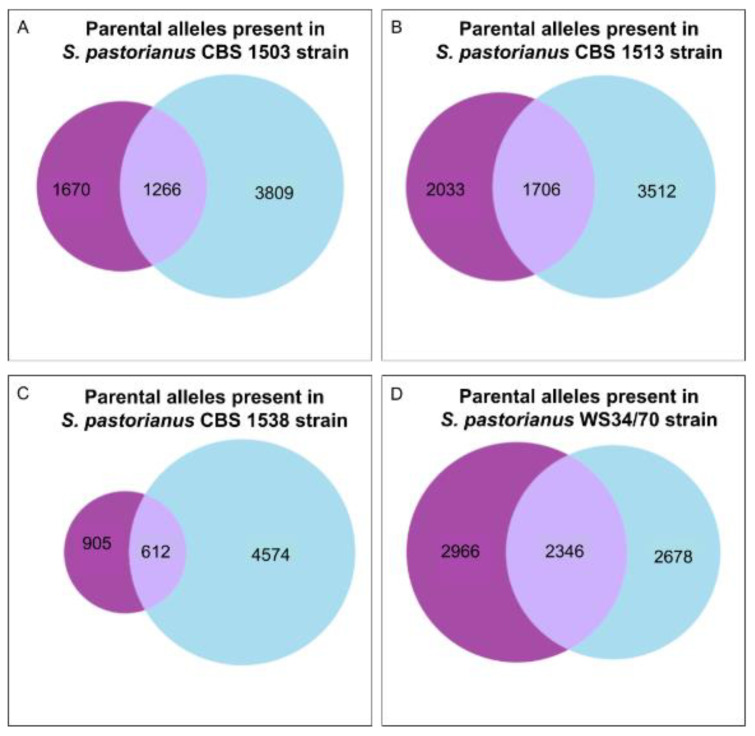
Venn diagrams showing the inherited *S. eubayanus-like* and *S. cerevisiae-like* parental alleles that have a unique function, represented in light blue and purple circle, respectively; and in the intersection those the parental ortholog alleles that share the same function. (**A**): *S. pastorianus* CBS1503. (**B**): *S. pastorianus* CBS1513. (**C**): *S. pastorianus CBS1538.* (**D**): *S. pastorianus* WS34/70.

We investigated the phylogenetic relationship of the inherited *S. cerevisiae*-like and *S. eubayanus*-like genes for the four strains of *S. pastorianus*. In total, there are 1266, 1706, 612, and 2346 pairs of *S. cerevisiae*-like and *S. eubayanus*-like genes that are one-to-one orthologs (and therefore share the same function) in the *S. pastorianus* CBS 1503, CBS 1513, CBS 1538, and WS 34/70 strains, respectively (Figure 7 and Supplementary Table S3). As expected, the almost tetraploid Group II strain (WS 34/70) has the higher number of redundant genes. Out of the three Group I *S. pastorianus* strains studied, CBS1538 (Figure 7C) shows the highest loss of the *S. cerevisiae* genome, and the lowest amount of redundant functional genes. Interestingly, the low amount of redundancy in this case comes primarily by the loss of the *S. cerevisiae*-like allele.

**Figure 8:**
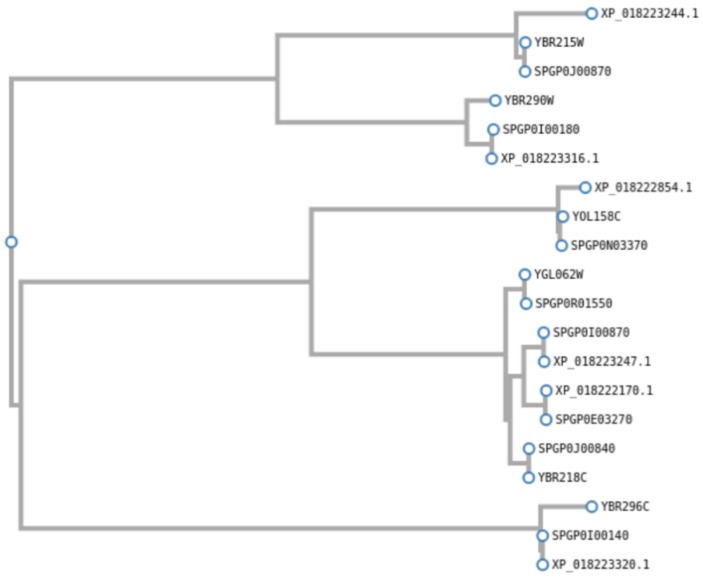
Phylogenetic tree of *S. pastorianus* WS34/70 genes with their homologs in *S. cerevisiae* S288C and *S. eubayanus* FM1318.

We also validated a sample of HybridMine output predictions for correct assignment of parental alleles and homologs by constructing gene trees. Using ClustalW, we aligned eight *S. pastorianus* WS 34/70 genes with their best homolog in *S. cerevisiae* and in *S. eubayanus*, including two pairs of paralogs (20 sequences in total). HybridMine correctly predicted the parental origin of all eight *S. pastorianus* genes and was able to tease apart the paralogs inherited from *S. cerevisiae* and *S. eubayanus* (Table 3 and Figure 8). In fact, *S. cerevisiae* pyruvate decarboxylase genes, *PYC1* (YGL062w) and *PYC2* (YBR218C) are paralogs, and similarly the *S. eubayanus* XP_018223247.1 and XP_018222170.1 are annotated in UniProtKB as pyruvate decarboxylase genes. HybridMine accurately predicted all four of them as homologs.

## 4. Discussion

Mining the genome of hybrid species is becoming of major importance to understand their evolution, adaptation processes and phenotypic landscape in relation to their environmental setting. Computational predictive approaches are also rarely employed due to the lack of molecular data on hybrid strains. The sequencing of large hybrids genome has now become more accurate with the development of the long-read third-generation sequencing technologies such as Nanopore [29]. Although general annotation tools are in place, a bioinformatic method that specifically predict the parental alleles in hybrid genomes is still lacking. The identification of parental alleles in hybrids is crucial to make accurate functional annotation, to assign sequences to the right biological function, to study *cis/trans* transcriptional regulation, and to inform how genetic redundancy of ortholog alleles impacts on phenotype. Overall, accurate annotation of hybrids facilitates the study of their evolution as most of them are still undergoing genome reduction to acquire stability. 

In the past two years, tools investigating hybrid genomes using short-read data sequencing have emerged. Their strategy consists in mapping short-read sequencing data of the hybrid genome of interest to a combined library of short-reads from both its parental species. The sppIDer pipeline [30] has been developed to specifically identify the coverage of each parental species in the hybrid and cannot carry out functional annotation. The MuLoYDH workflow [31] has been created with the aim of tracking mutational landscape in yeast diploid hybrids, and therefore is designed to primarily determine SNPs, SNVs, and loss-of-heterozygosity. This tool is computationally intensive and is not intended for functional annotation of coding regions in hybrids.

Given the complex nature of the hybrid genomes, it is difficult with standard bioinformatic tools to discriminate parental alleles, orthologs genes, and correctly identify the homologs, including paralogs. Established annotation tools Blast2go and eggNOG-mapper, although they are efficient to annotate organisms in general as they rely in homology search, as HybridMine, their design is not adapted for predicting parental alleles in hybrid genomes. eggNOG-mapper does not restrain the ortholog search to the parental genomes; therefore, its output is highly inaccurate for parental allele prediction. Blast2GO is more adapted for that purpose compared to eggNOG-mapper as the user can restraint the homology search to parental organisms. However, the output is not fully accurate as young paralogs are annotated with the same best homolog, and one-to-one orthologs are not considered. Furthermore, both tools are not taking into account aneuploidy that often occurs in hybrid genomes. When the parental origins are known, HybridMine is the most suitable tool as it searches for one-to-one orthologs between the hybrid and the parents, and it identifies the group of homologs and paralogs in the hybrid genome. HybridMine requires as an input at least three FASTA format files containing all the gene sequences of the hybrid and the two parental genomes. HybridMine is also supporting up to four parental genomes as input. The increase in the time cost when running three or four parental genomes is linear rather than exponential, and it is still less than 1 min for four parental genomes with a size of ca. 6000 genes each.

As HybridMine is using BLAST algorithm to align the sequences, it is preferable to be consistent in the data type. If a gene sequence includes introns and UTRs, the input of the hybrid and parents sequence for such gene should be the same (i.e., all including the UTRs and Introns, or all omitting them).

## 5. Conclusions

We developed HybridMine, a new fast tool that can be used to reliably map orthologs and paralogs of any hybrid organisms of known parental species. As proof of principle, we benchmarked our pipeline against two established annotation tools and we functionally annotated the genomes of four different yeast hybrids of industrial importance. While the sequence of *S. cerevisiae* genome has been available for almost 25 years, only recently yeast hybrid genomes have been sequenced [18,19,20,32,33,34], creating the need for new bionformatics tools to investigate the complex aneuploid composition of their genomes. Allele predictions in hybrids will allow accurate comparative genomic analyses (including studies on gene retention and gene loss and transcriptional plasticity) and accurate in silico design of new advantageous strains for biotechnological purposes. Specifically, we used HybridMine to functionally annotate (and made the data available to the public for the first time) four *S. pastorianus* yeast strains used for lager-style beer production, which are currently targeted to be improved in the beer industry. In summary, HybridMine is an unmatched fast and accurate annotation tool for hybrid genomes with known parental origins. HybridMine is available at https://github.com/Sookie-S/HybridMine.

## Figures and Tables

**Table 1:** Comparison between eggNOG-mapper, Blast2GO, and HybridMine. NA refers to a feature that is not provided within the tool. Green and red shaded areas highlight desirable and missing aspects of the tool.

	Parental Genomes	Prediction	eggNOG-Mapper	Blast2GO	HybridMine
*S. pastorianus WS34/70*	*S. cerevisiae*	Homologs	NA	5373	8182
		1:1 Orthologs	9997	NA	5312
		Paralogs	NA	NA	1738
	*S. eubayanus*	Homologs	NA	6148	8222
		1:1 Orthologs	0	NA	5024
		Paralogs	NA	NA	1586
			Search only wide taxa group	Search specific to the parental genomes	Search specific to the parental genomes
			Free tool	Tool under license	Free tool

**Table 2:** HybridMine predictions of the total numbers of *S. cerevisiae-*like and *S. eubayanus*-like alleles among the total number of genes.

*S. pastorianus* Strain Annotated in This Study	CBS 1538	CBS 1503	CBS 1513	WS 34/70
**Total Number of Genes**	7288	8714	9728	11,265
**Number of Predicted Alleles in *S. eubayanus***	5186	5075	5218	5024
**Number of Predicted Alleles in *S. cerevisiae***	1517	2936	3739	5312
**1:1 Orthologs with Same Sequence Identity in Both Parents**	9	10	9	7
**ORFs with less than 80% Sequence Identity With a Predicted Parental Allele**	9	3	4	2
***S. pastorianus* Genes With no Parental Allele Predicted**	567	690	758	920
**Number of Different Paralogs**	2814	3056	3316	3652

**Table 3:** HybridMine parental allele prediction output for eight *S. pastorianus* WS 34/70 genes and parental allele phylogenetic tree output.

*S. pastorianus* WS 34/70 Strain Genes	Best Homolog in *S. cerevisiae*	Best Homolog in *S. eubayanus*	Parental Allele Prediction with HybridMine	Parental Allele Phylogenetic Tree Output
SPGP0N03370	YOL158C	XP_018222854.1	YOL158C	YOL158C
SPGP0I00140	YBR296C	XP_018223320.1	XP_018223320.1	XP_018223320.1
SPGP0I00180	YBR290W	XP_018223316.1	XP_018223316.1	XP_018223316.1
SPGP0J00870	YBR215W	XP_018223244.1	YBR215W	YBR215W
SPGP0J00840	YBR218C	XP_018223247.1	YBR218C	YBR218C
SPGP0R01550	YGL062W	XP_018222170.1	YGL062W	YGL062W
SPGP0I00870	YBR218C	XP_018223247.1	XP_018223247.1	XP_018223247.1
SPGP0E03270	YGL062W	XP_018222170.1	XP_018222170.1	XP_018222170.1

## Supplementary Materials

The following are available online at https://www.mdpi.com/2076-2607/8/10/1554/s1, Table S1: Link to the genomic sources. Table S2: Group of homolog genes in S. cerevisiae S288C genome predicted by HybridMine, and their UniProt annotation. Table S3: S. cerevisiae-like and S. eubayanus-like genes that are one-to-one orthologs (and therefore share the same function) in the S. pastorianus CBS 1503, CBS 1513, CBS 1538, and WS 34/70 strains. File S1: Saccharomyces pastorianus CBS 1503 parental alleles and paralogs prediction. File S2: Saccharomyces pastorianus CBS 1513 parental alleles and paralogs prediction. File S3: *Saccharomyces pastorianus* CBS 1538 parental alleles and paralogs prediction. File S4: *Saccharomyces pastorianus* CBS WS34/70 parental alleles and paralogs prediction
